# Utilizing Co^2+^/Co^3+^ Redox Couple in P2‐Layered Na_0.66_Co_0.22_Mn_0.44_Ti_0.34_O_2_ Cathode for Sodium‐Ion Batteries

**DOI:** 10.1002/advs.201700219

**Published:** 2017-07-06

**Authors:** Qin‐Chao Wang, Enyuan Hu, Yang Pan, Na Xiao, Fan Hong, Zheng‐Wen Fu, Xiao‐Jing Wu, Seong‐Min Bak, Xiao‐Qing Yang, Yong‐Ning Zhou

**Affiliations:** ^1^ Department of Materials Science Fudan University Shanghai 200433 P. R. China; ^2^ Chemistry Division Brookhaven National Laboratory Upton NY 11973 USA; ^3^ Department of Chemistry & Laser Chemistry Institute Fudan University Shanghai 200433 P. R. China

**Keywords:** cathode materials, layered structures, sodium‐ion batteries, X‐ray absorption spectroscopy

## Abstract

Developing sodium‐ion batteries for large‐scale energy storage applications is facing big challenges of the lack of high‐performance cathode materials. Here, a series of new cathode materials Na_0.66_Co*_x_*Mn_0.66–_ *_x_*Ti_0.34_O_2_ for sodium‐ion batteries are designed and synthesized aiming to reduce transition metal‐ion ordering, charge ordering, as well as Na^+^ and vacancy ordering. An interesting structure change of Na_0.66_Co*_x_*Mn_0.66–_ *_x_*Ti_0.34_O_2_ from orthorhombic to hexagonal is revealed when Co content increases from *x* = 0 to 0.33. In particular, Na_0.66_Co_0.22_Mn_0.44_Ti_0.34_O_2_ with a P2‐type layered structure delivers a reversible capacity of 120 mAh g^−1^ at 0.1 C. When the current density increases to 10 C, a reversible capacity of 63.2 mAh g^−1^ can still be obtained, indicating a promising rate capability. The low valence Co^2+^ substitution results in the formation of average Mn^3.7+^ valence state in Na_0.66_Co_0.22_Mn_0.44_Ti_0.34_O_2_, effectively suppressing the Mn^3+^‐induced Jahn–Teller distortion, and in turn stabilizing the layered structure. X‐ray absorption spectroscopy results suggest that the charge compensation of Na_0.66_Co_0.22_Mn_0.44_Ti_0.34_O_2_ during charge/discharge is contributed by Co^2.2+^/Co^3+^ and Mn^3.3+^/Mn^4+^ redox couples. This is the first time that the highly reversible Co^2+^/Co^3+^ redox couple is observed in P2‐layered cathodes for sodium‐ion batteries. This finding may open new approaches to design advanced intercalation‐type cathode materials.

## 1 Introduction

Lithium‐ion batteries (LIBs) have attracted intense attention as energy sources for portable electronic devices due to their high energy and power densities. However, because of the low abundance of lithium in the Earth's crust and nonuniform geographic distribution, their further application in the large‐scale energy storage for renewable energy systems, such as solar/wind power and smart grid, faces significant challenges.^1^, ^2^, ^3^ Alternatively, with the similar intercalated electrochemistry to LIBs, sodium‐ion batteries (SIBs) have been considered as a promising alternative for large‐scale energy storage application due to the nature abundance and low cost of sodium.^4^

However, the lack of high‐performance cathode materials is one of the major obstacles for developing high‐performance room temperature SIBs.^5^, ^6^, ^7^, ^8^ Over the past years, various cathode materials have been investigated, including layered oxides,^9^, ^10^, ^11^, ^12^, ^13^, ^14^, ^15^ tunnel‐structured oxides,^16^, ^17^, ^18^ polyanion compounds,^19^, ^20^ and organic compounds.^21^ Among them, layered oxides are promising cathode materials with a general formula of Na*_x_*TMO_2_ (TM = transition metals, such as Co, Ni, Mn, Cr, Fe, and Cu),^9^, ^12^, ^22^, ^23^, ^24^ which can be categorized into two main groups: P2‐type and O3‐type. P2 and O3 are defined according to the oxygen stacking sequence and coordination environment of the alkali ions.^25^, ^26^ Generally, O3‐type compounds have higher reversible capacity compared with P2‐type compounds, but their cycling stability is not good and many of them are not stable in air.^9^, ^27^, ^28^ In contrast, the P2‐type layered cathode materials usually deliver better cycle performance and rate capability due to the large trigonal prismatic sites occupied by Na^+^ which is beneficial for Na‐ion transportation.^25^ Mn‐based P2‐type cathode materials are promising for their good performance and low cost. P2‐Na_0.67_MnO_2_ can deliver a high capacity of 175 mAh g^−1^, but suffers from poor cycle stability during sodiation/desodiation due to the presence of Jahn–Teller active Mn^3+^,^29^ which is a serious problem for practical implementation of such materials.^30^, ^31^ In addition, the existence of Mn^3+^ may produce Mn^2+^ ions due to disproportionation reaction, which favor to dissolve into the carbonate‐based electrolytes.^32^, ^33^ To address these issues, using other transition metals, such as cobalt, nickel to substitute the Mn ions in layered Na*_x_*MnO_2_, has been investigated.^29^, ^30^, ^31^ It was found that P2‐type Na_2/3_Co_2/3_Mn_1/3_O_2_ displays a reversible 0.5 Na^+^ intercalation between 1.5 and 4.0 V. The good reversibility is attributed to the fact that the introduced low‐spin Co^3+^ ions can stabilize Mn ions at a valence state of 4+.^23^, ^34^ Li et al. reported that Ni and Co substitution in Na_0.7_Mn_0.7_Ni_0.3−_ *_x_*Co*_x_*O_2_ could enhance sodium diffusion coefficient and high‐rate capability by shortening the bond lengths of TM—O and O—O.^12^ In addition, introducing Ti ions into transition metal layer can reduce the structural distortions during charge and discharge processes and suppress the formation of a secondary rock salt phase at high voltage charge.^10^, ^22^

It has been reported by Hu et al. that there are three types of ordering in the structure of P2‐layered Na*_x_*[M1,M2]O_2_ oxides which could have negative effects on the electrochemical performance: transition metal ordering (different transition metal ions prefer to occupy different atomic sites, rather than indistinguishable occupation), charge ordering (preferred site occupations of ions with different charge, such as M^3+^/M^4+^ ordering), and Na^+^/vacancy ordering.^35^ In a large number of P2‐type layered oxides,^12^, ^22^, ^23^, ^24^, ^36^, ^37^, ^38^ transition metal ordering is mainly determined by the difference between the ionic radii of M1 and M2, in which small difference (usually less than 15%) favors to form disordered arrangement if M1 and M2 content is close to a rational rate, while a larger difference (greater than 15%) favors an ordered arrangement. In the case of charge ordering, it is determined by the redox potential of M1 and M2. Small difference of redox potential is favorable for charge ordering. In a P2‐type layered transition metal oxides, if these three kinds of ordering can be effectively suppressed, good electrochemical performance can be expected.

Therefore, in this study, we designed a material using the transition metal ions with very similar ionic radii and redox potentials in order to construct a P2‐type layered Na_0.66_Co*_x_*Mn_0.66−_ *_x_*Ti_0.34_O_2_ (0 ≤ *x* < 0.33) compound aiming to suppress the transition metal, charge and Na^+^/vacancy ordering. It is expected that the cosubstitution of Co and Ti can enhance the rate capability and reduce the structural distortion.

## 2 Results and Discussion

**Figure 1:**
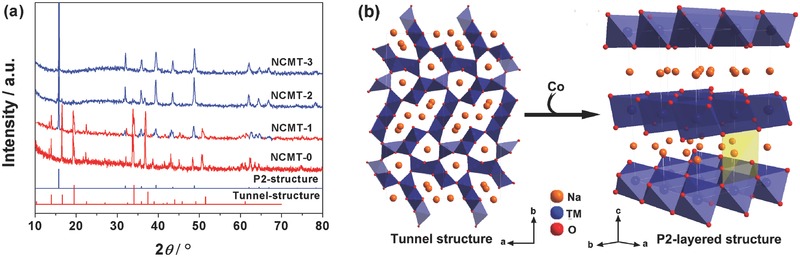
a) X‐ray diffraction patterns of as‐prepared NCMT‐0, NCMT‐1, NCMT‐2, and NCMT‐3 (red and blue lines are indexed to tunnel and P2‐layered structure, respectively). b) A schematic of the evolution of NCMT structure from tunnel structure to P2‐layered structure with increasing Co substitution. The red, violet, and yellow balls stand for oxygen, transition metal, and sodium ions, respectively.

A series of air stable Na_0.66_Co*_x_*Mn_0.66−_ *_x_*Ti_0.34_O_2_ (*x* = 0, 0.11, 0.22, and 0.33), denoted as NCMT‐0, NCMT‐1, NCMT‐2, and NCMT‐3, respectively, are synthesized by a conventional solid‐state reaction. The X‐ray diffraction (XRD) pattern of NCMT‐0 shown in the **Figure** **1**a can be indexed by a typical tunnel‐type structure with a spacing group of Pba.^18^ When 0.11 Mn is substituted by Co to obtain NCMT‐1, a P2‐type layered phase is observed, coexisting with the tunnel structure, as shown in XRD pattern of NCMT‐1 in Figure 1a. When the content of Co increased to 0.22 (NCMT‐2), diffraction peaks of the tunnel structure are completely disappeared, showing a pure P2‐type structure, which is isostructural with the typical P2‐type Na_0.67_Ni_0.33_Mn_0.67_O_2_ (JCPDS No. 54‐0894). When the Co content is further increased to 0.33 in NCMT‐3, no more structure changes are observed. These results suggest a structural change from tunnel structure to P2‐type layered structure when the amount of Co substitution of Mn in NCMT is increased from 0 to 0.22, as shown in Figure 1b.

The scanning electron microscopy (SEM) images of the synthesized NCMT‐2 and NCMT‐3 with P2‐layered structure (Figures S1 and S2, Supporting Information) exhibit a sheet‐like morphology. It can be seen that the as‐synthesized samples are composed of large irregular sheets in a size range of 3–5 µm. The Energy dispersive X‐ray spectroscopy mapping of NCMT‐2 and NCMT‐3 exhibits homogeneous distributions of elements Na, Co, Mn, Ti, and O in these sheets. The stoichiometry of the as‐synthesized NCMT‐2 and NCMT‐3 shown in Table S1 in the Supporting Information is close to 0.22:0.44:0.34, and 0.33:0.33:0.34 for Co, Mn, and Ti, respectively, agreed well with the designed composition of NCMT‐2 and NCMT‐3.

**Figure 2:**
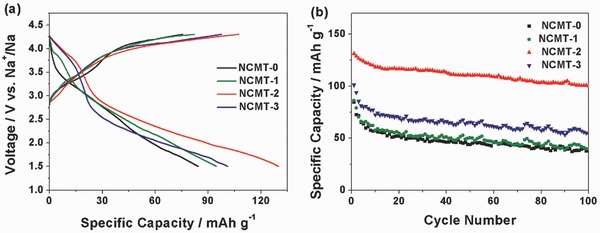
a) Galvanostatic charge/discharge curves of NCMT‐0 (black), NCMT‐1 (blue), NCMT‐2 (red), and NCMT‐3 (green) in the initial cycle at a current rate of 0.1 C (17.7 mA g^−1^) in the potential range of 1.5–4.3 V versus Na^+^/Na. b) The long‐term cycle performance of the synthesized NCMT‐*x* electrodes at a current density of 0.2 C.

The electrochemical properties of the synthesized NCMT‐*x* (*x* = 0, 1, 2, and 3) are characterized by galvanostatic charge/discharge measurements. **Figure** **2**a shows the first charge and discharge curves of NCMT‐*x* in the voltage range of 1.5–4.3 V. The capacities of NCMT‐*x* (*x* = 0, 1, 2, and 3) during the first charge are 75, 95, 107, and 82 mAh g^−1^, respectively. In the discharge process, NCMT‐2 shows a higher specific capacity of 131 mAh g^−1^, comparing with NCMT‐0, NCMT‐1, and NCMT‐3. Figure 2b shows the cycle performance of the four NCMT electrodes in 100 cycles. It can be seen that NCMT‐2 maintains a reversible capacity of 104 mAh g^−1^ when cycled at 0.2 C rate, while the others show quite low capacities of less than 70 mAh g^−1^ after 100 cycles. It implies that a Co content of 0.22 in NCMT is the optimized value for the reversible specific capacity and the capacity retention.

**Figure 3:**
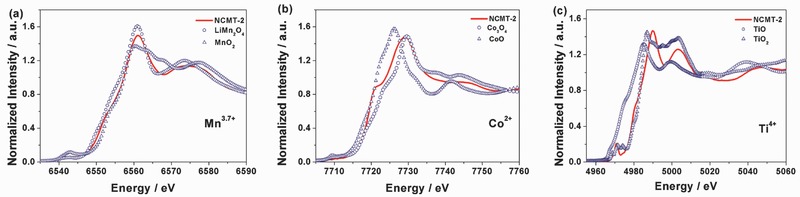
XANES spectra of the a) Mn, b) Co, and c) Ti K‐edges of pristine NCMT‐2 and corresponding metal oxide references.

Ex situ X‐ray absorption spectroscopy (XAS) was carried out to provide an in‐depth understanding of the local structure and the valence state of transition metals in NCMT‐2. **Figure** **3** illustrates the X‐ray absorption near edge structure (XANES) spectra of Co, Mn, and Ti K‐edge. By comparing with the spectra of the standard transition metal oxide compounds, it is revealed that the average valence states of the Co, Mn, and Ti ions in the as‐synthesized NCMT‐2 are 2+, 3.7+, and 4+. It is very surprising that Co ions can be stabilized at 2+ in the P2‐type layer‐structured compounds, since the valence state of Co ions in most layer‐structured cathode materials is 3+.^12^, ^23^, ^39^ In addition, the valence state of Mn ions in NCMT‐2 is 3.7+, which is higher than Mn^3+^ in tunnel‐structured Na_0.66_Mn_0.66_Ti_0.34_O_2_.^18^ It is well known that high‐spin Mn^3+^ with the electron configuration of (t_2g_)^3^(e_g_)^1^ in the 3D orbital exhibits very strong Jahn–Teller distortion due to the single occupancy of a degenerate e_g_ orbital.^12^, ^22^ The low valence Co^2+^ substitution in NCMT‐2 introduces more Mn^4+^ because the required charge balance, which resulted the average Mn^3.7+^ in the P2‐type structure. Such increased average valence state at Mn^3.7+^ is effectively suppressed the Jahn–Teller distortion and stabilized the structure by perturbing the magnetic arrangement.^12^, ^40^ In addition, the decreasing of Mn^3+^ concentration in the cathode may help to mitigate Mn dissolution from the cathode into the electrolyte. Considering this unique charge distribution between the transition metal ions, it is intriguing to know how the low valence Co^2+^ in NCMT‐2 contributes to the charge compensation during charge/discharge processes and whether it is reversible.

The magnitudes of the Fourier transformed extended X‐ray absorption fine structure (FT‐EXAFS) spectra and their least square fits are plotted in the Figure S3 in the Supporting Information. The first peak at *R* ≈ 2.0 Å is due to the contribution of the nearest TM–O_6_ octahedra, while the second peak at *R* ≈ 3.0 Å is attributed to the TM–TM_6_ hexagon on the a–b plane in the second coordination shell. It should be noted that the FT‐EXAFS spectra are not phase corrected; therefore, the actual bond lengths are ≈0.3–0.4 Å longer.^41^, ^42^, ^43^ Structure parameters derived from the fitting are listed in Table S2 in the Supporting Information. It is shown that both Mn–O and Mn–TM distances are shorter than the distances of Co–O and Co–TM, which agrees well with the larger ion radii of Co^2+^ than that of Mn^3.7+^.

**Figure 4:**
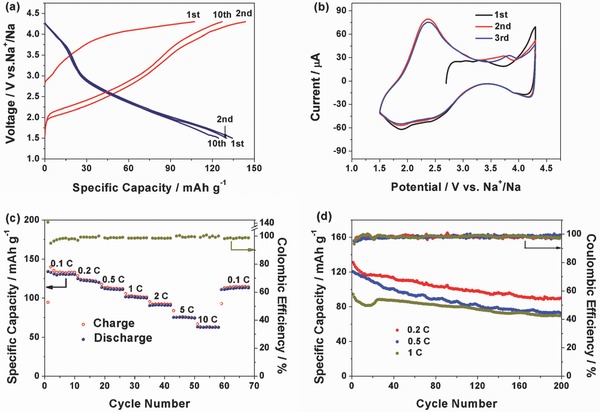
a) Galvanostatic charge/discharge curves of NCMT‐2 in the initial ten cycles at a current rate of 0.1 C (17.7 mA g^−1^) in the potential range of 1.5–4.3 V versus Na^+^/Na. b) The initial two cyclic voltammograms (CV) of the NCMT‐2 electrode between 1.5 and 4.3 V versus Na^+^/Na at a scan rate of 0.3 mV s^−1^. c) Rate capability at various current rates from 0.1 to 10 C. d) Cycling performance at 0.2, 0.5, and 1 C for 200 cycles.

The electrochemical performance of NCMT‐2 in sodium cells was studied in details by galvanostatic charge/discharge and cyclic voltammetry (CV) measurements shown in **Figure** **4**. As shown in Figure 4a, the open‐circuit voltage of the NCMT‐2/Na cell is close to 2.75 V (vs Na^+^/Na). During the initial charge process, a slope curve from 2.7 to 3.8 V followed by a plateau at around 4.1 V is observed. A charge capacity of 107 mAh g^−1^ is obtained, which corresponds to about 0.40 Na extracted per NCMT‐2. During the discharge process, two slopes from 4.3 to 3.0 V and 3.0 to 1.5 V are observed. The initial discharge capacity is about 131 mAh g^−1^, corresponding to 0.49 Na intercalated per NCMT‐2. The voltage curve of the second charge is much different from the initial charge. A low average voltage is observed and a large capacity of 140 mAh g^−1^ is obtained. The difference could be due to the change of the redox couples between the initial charge and the second one. In the subsequent cycles, the shapes of the voltage curves are quite similar as the second cycle, indicating good reversibility of the subsequent cycles.

The typical CV curves of the NCMT‐2 electrode in the first three cycles are presented in Figure 4b. Two pairs of redox peaks at 2.3 and 4.0 V (vs Na^+^/Na) are observed. They can be assigned to the complex redox processes of Co and Mn with mixed valence states,^12^, ^44^ since the redox potential of Ti ions is usually below 1.5 V.^35^ The polarization of charge and discharge is relatively low compared with many other P2‐layered cathode materials for SIBs.^35^, ^44^, ^45^ After the initial charge process, the CV curves are overlapped very well, indicating high reversibility. These results are in good agreement with the galvanostatic charge/discharge data in Figure 4a.

Figure 4c shows the rate capacity of the NCMT‐2 electrode versus cycle life. The cells were charged and discharged at various rates from 0.1 to 10 C. The NCMT‐2 electrode delivers the reversible capacities of 134.1, 124.9, 113.7, 101.8, 90.7, 75.6, and 63.2 mAh g^−1^ at the current rates of 0.1, 0.2, 0.5, 1, 2, 5, and 10 C (C rate is calculated based on a specific capacity of 177 mAh g^−1^), respectively, suggesting good rate capability. When the current rate is reduced back to 0.1 C, the NCMT‐2 electrode can recover a capacity of 113.6 mAh g^−1^, implying good reversibility of NCMT‐2 under a wide current range. Figure 4d illustrates the long‐term cyclic performance of NCMT‐2 at 0.2, 0.5, and 1 C rates. It can be seen that reversible capacities of 71.5 and 69.7 mAh g^−1^ can be retained after 200 cycles, slightly higher than reported tunnel‐type Na_0.66_Mn_0.66_Ti_0.34_O_2_.^18^ The long‐term cycling performance of NCMT‐2 is not quite satisfactory, which could be improved through doping or decreasing the particle size.

**Figure 5:**
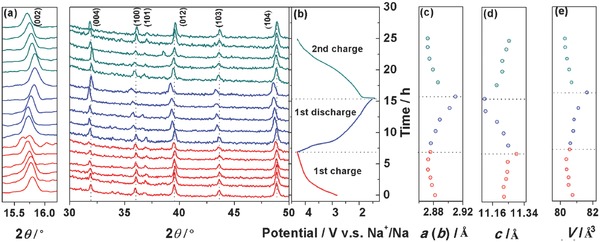
Structural evolution of NCMT‐2 during the electrochemical cycles. a) Ex situ X‐ray diffraction patterns collected during the first charge/discharge and the second charge of the Na/NCMT‐2 cells at a current rate of 10 C/10 in the potential range between 1.5 and 4.3 V. b) Corresponding charge/discharge profile. c,d) Lattice parameters of *a* (*b*) and *c* in hexagonal crystal at different charge and discharge states calculated form the XRD results. e) Unit cell volume of NCMT‐2 during charge and discharge.

To monitor the structural evolution of the NCMT‐2 electrode during charge and discharge processes, ex situ XRD experiments are performed at various charge/discharge states as shown in the **Figure** **5**a. Corresponding voltage curves are shown on the right side in Figure 5b. In the first charge process from 2.75 to 4.05 V (vs Na^+^/Na), the (002) peak continuously shifts to lower 2θ angle, suggesting a solid‐solution reaction mechanism with the *c*‐axis expansion. It is consistent with the sloppy charge/discharge curve during the first charge. When the cell is further charged to 4.3 V, the (002) peak splits into two peaks, indicating new phase formation during the high voltage charge possibly due to the formation of O^2−^ vacancy.^46^ In the first discharge process, the two split (002) peaks merged to a single peak in the early stage of discharge, then continue shifting to higher 2θ angles, indicating *c*‐axis shrinking. At the end of discharge, the (002) peak returns to the same position as the pristine NCMT‐2, suggesting a reversible structure change. In the second charge process, the phase evolution behavior is in the same pathway as the initial charge, but no peak splitting is observed at the end of charge, implying good structure stability. On the other hand, unlike (003) peak, which only reflects the change of the lattice parameter “*c*”, the (100) peak shifts to higher angles during charge process, and returns to the original position during discharge process, indicating a reverse change of lattice parameters “*a*” and “*b*”. The detailed evolution of lattice parameters and unit cell volume are shown in the Figure 5c–e. The unit cell breathing behavior of NCMT‐2 during charge and discharge follows the typical pathway of layer‐structured cathode materials.^47^

**Figure 6:**
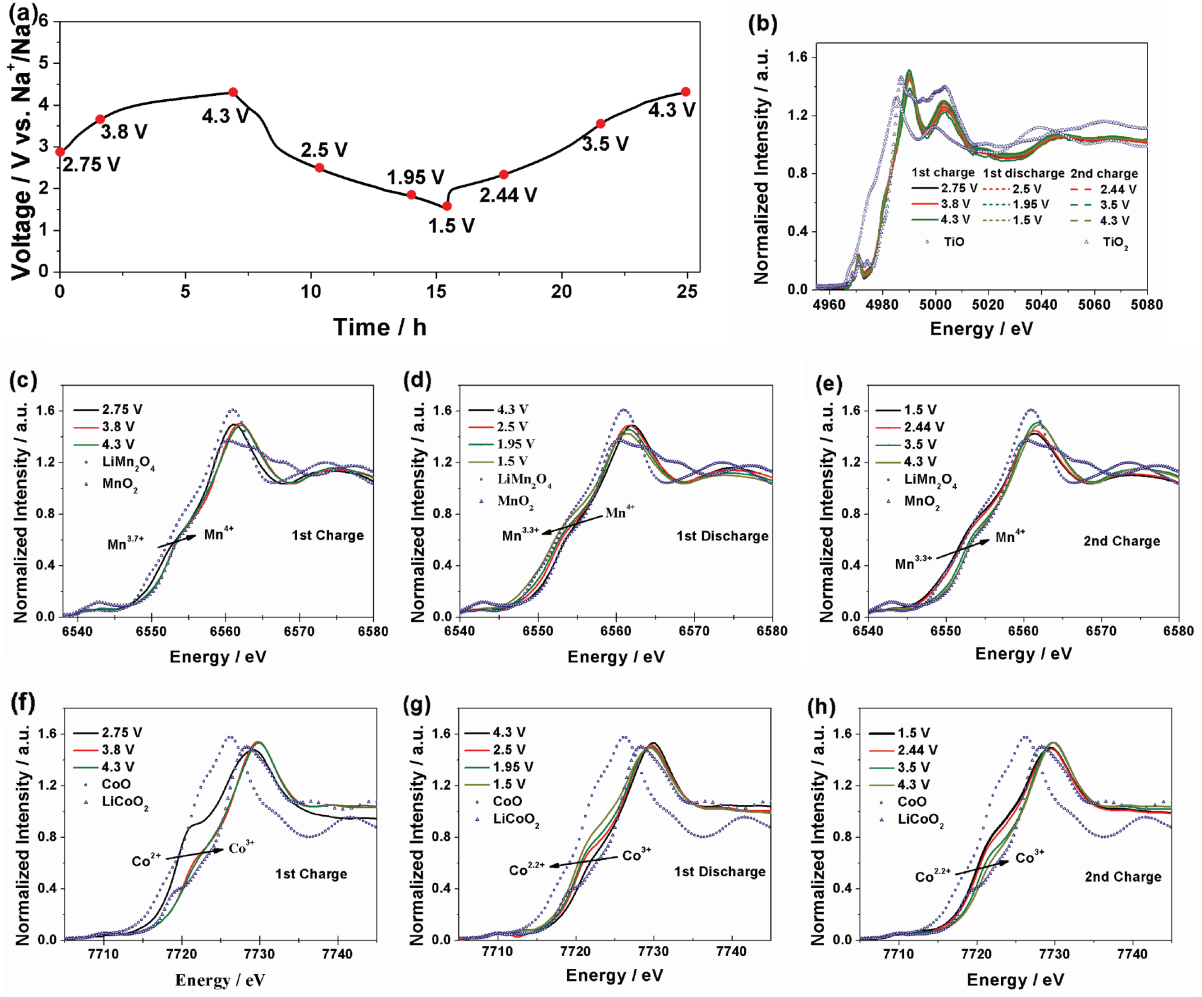
a) Charge and discharge plots of NCMT‐2 electrode for ex situ XAS measurement. b) Ti K‐edges XANES of NCMT‐2 at various stages during the first charge/discharge and the second charge processes; c) Mn and f) Co K‐edges XANES of NCMT‐2 at various stages during the first charge; d) Mn and g) Co K‐edges XANES of NCMT‐2 at various stages during the first discharge; e) Mn and h) Co XANES of NCMT‐2 at various stages during the second charge.

The changes of the local structure and valence state of the NCMT‐2 during the charge/discharge processes are investigated by ex situ XAS at the Co, Mn, and Ti K‐edge. **Figure** **6** shows the XANES spectra of Mn, Co, and Ti during the first charge, first discharge, and second charge processes, respectively. The charge/discharge curves and the corresponding states where the XAS scans are taken are shown in Figure 6a. For Ti K‐edge spectrum (Figure 6b), it can be seen that the edge position has no shift during the charge and discharge processes, indicating very stable Ti^4+^ is maintained with no valence change during sodium intercalation and deintercalation. However, for Mn and Co K‐edge spectra in the initial charge (Figure 6c,d), the white line shifts to higher energy, indicating Co and Mn cations are oxidized to higher valence states during the charge process. Compared with the standard references, it can be roughly estimated that the valence states of Co and Mn in NCMT‐2 at the fully charge state are around 3+ and 4+. If we calculate the charge compensation during initial charge based on the valence change of transition metal ions (Co^2+^→Co^3+^, Mn^3.7+^→Mn^4+^), the theoretic capacity of initial charge should be 93.6 mAh g^−1^. However, the actual capacity is 107 mAh g^−1^, suggesting that oxygen may possibly contribute to the charge compensation by forming O^2−^ vacancy, associating with (002) peak splitting in Figure 4a.

In the first discharge process (Figure 6e,f), the K‐edge of both Co and Mn shifts back toward lower energy. It can be estimated that Mn^4+^ is reduced back to Mn^3.3+^, corresponding to 0.31 Na^+^ intercalated due to Mn reduction. However, Co^3+^ cannot be reduced back to Co^2+^ completely. An average valence state of 2.2+ for Co ions is reached after discharge to 1.5 V, corresponding to 0.18 Na^+^ intercalated. Therefore, a total number of 0.49 Na^+^ per NCMT‐2 is extracted during discharge. It agrees well with the initial discharge capacity of 131 mAh g^−1^. In the second charge process, both of XANES spectra of Mn and Co undergo reversed changes compared to the initial discharge process, suggesting a reversible evolution of the electronic structure and surrounding environment of Mn and Co in NCMT‐2. The FT‐EXAFS spectroscopy during charge and discharge is shown in Figure S4 in the Supporting Information. The structural parameters extracted from fitting these spectra are listed in Tables S3 and S4 in the Supporting Information. It can be seen that the changes of the first coordination shell (Co–O) and second one (Co–TM) around Co are much significant than those around Mn, further confirms the predominant contribution of Co to the electrochemical reactions. To the best of our knowledge, this is the first time that the highly reversible Co^2+^/Co^3+^ redox couple is revealed in P2‐layered cathodes for SIBs experimentally, while most of Co‐contained layer‐structured cathodes utilize the Co^3+^/Co^4+^ redox couple for charge compensation such as the well‐known layered LiCoO_2_ and NaCoO_2_. In addition, the diffused Ti 3D orbitals can create strong Ti–TM interaction between two adjacent edge sharing TM–O_6_ and Ti–O_6_ octahedra, so introducing Ti in this system might be able to suppress the Mn migration by changing the local electronic structure. The low valence Co^2+^ substitution results the formation of Mn^3.7+^, avoiding the strong Jahn–Teller distortion of Mn^3+^ thus stabilizing the P2‐layered structure. This comprehensive design is beneficial for improving electrochemical performance of Mn‐contained layered cathode materials.

## 3 Conclusion

In summary, we synthesized a series of Na_0.66_Co*_x_*Mn_0.66−_ *_x_*Ti_0.34_O_2_ (*x* = 0, 0.11, 0.22, and 0.33) cathode materials for SIBs. With the increase of Co content, the crystal structure of the as‐synthesized materials changed from tunnel‐type to P2‐type layered structure. Among them, NCMT‐2 delivers the highest reversible capacity of 120 mAh g^−1^ at 0.1 C rate. A reversible capacity of 63.2 mAh g^−1^ at 10 C rate can be obtained, indicating a promising rate capability. X‐ray diffraction results reveal a single‐phase reaction pathway of NMCT‐2 during charge and discharge processes in the voltage range of 1.5–4.3 V. XAS results suggest that two redox couples Co^2.2+^/Co^3+^ and Mn^3.3+^/Mn^4+^ contribute to the charge compensation during charge and discharge. It should be noted that this is the first time to observe the redox couple of Co^2+^/Co^3+^ in the P2‐type layered oxides. The low valence Co^2+^ substitution is responsible for the average valence state of Mn at Mn^3.7+^, effectively suppressing the Mn^3+^‐induced Jahn–Teller distortion, which in turn strengthening the P2‐type layered structure. Ti substitution could smooth the charge/discharge plateaus effectively, and contribute to suppress transition metal migration. The utilization of Co^2+^/Co^3+^ redox couple in layer‐structured cathode systems may open new approaches to design high‐performance cathode materials for SIBs.

## 4 Experimental Section

*Material Synthesis*: Na_0.66_Co*_x_*Mn_0.66−_ *_x_*Ti_0.34_O_2_ (0 ≤ *x* ≤ 0.33) were synthesized by the simple solid‐state reaction method using Na_2_CO_3_, C_4_H_6_O_4_Co·2H_2_O, Mn_2_O_3_, and TiO_2_ as precursors in stoichiometric proportion with an excess of 5 mol% Na_2_CO_3_. After ball‐milled in acetone for 12 h, the mixture was dried overnight in an oven. The homogenous precursors were heated at 900 °C for 20 h in air.

*Material Characterization*: The morphology of the synthesis materials was characterized by field emission SEM (Cambridge S‐360). Powder XRD patterns were collected on an X‐ray diffractometer (BrukerD8 Advance, Germany) with Cu‐K*a* radiation (λ = 0.1540 nm) at 40 kV, 40 mA. XAS was performed at beamline 12BM of the advanced photon source at Argonne National Laboratory and beamline BL14W1 of the Shanghai Synchrotron Radiation Facility. Co, Mn, and Ti K‐edge XAS spectra were collected in transmission mode. The XAS data were processed using Athena and Artemis software packages.^48^, ^49^

*Electrochemical Performance*: The working electrode was prepared by spreading the slurry (70 wt% active materials, 20 wt% carbon black, and 10 wt% polyvinylidenefluoride (Sigma‐Aldrich) and an appropriate amount of *N*‐methyl‐2‐pyrrolidone (NMP)) onto aluminum foils. The electrodes were dried at 70 °C under vacuum for 12 h.

The electrochemical properties of the fabricated electrodes were evaluated in coin‐type cells assembled in an argon‐filled glove box. The active material loading of the working electrode is about 3.1 mg cm^−2^. In half cells, pure Na foil was used as counter electrode and a glass fiber (GB‐100R, ADVANTEC Co., Japan) as separator. The electrolytes solution was 1 m NaClO_4_ in a nonaqueous solution of ethylene carbonate/diethyl carbonate (EC: DEC, 1:1 in volume). Galvanostatic charge/discharge measurements were carried out on a Land CT2001A battery test system (Wuhan, China) in a voltage range from 1.5 to 4.3 V at 25 °C. The current densities and specific capacities of electrodes were calculated on the basis of the weight of the active materials. CV and electrochemical impedance spectroscopy test were performed on an electrochemical workstation (CHI 660E).

## Conflict of Interest

The authors declare no conflict of interest.

## Supporting information

Supplementary
