# Supplementary material for: Utilizing Co2+/Co3+ Redox Couple in P2‐Layered Na0.66Co0.22Mn0.44Ti0.34O2 Cathode for Sodium‐Ion Batteries
Source: Adv Sci (Weinh). 2017 Jul 6;4(11):1700219. doi: 10.1002/advs.201700219 (PMC5700635; doi:10.1002/advs.201700219)
Supplement: Supplementary file 1 — Supplementary [file ADVS-4-na-s001.pdf]

Copyright WILEY-VCH Verlag GmbH & Co. KGaA, 69469 Weinheim, Germany, 2017.

ADVANCED SCIENCE Open Access

Supporting Information

for *Adv. Sci.,* DOI: 10.1002/advs.201700219

Utilizing Co^{2+}/Co^{3+} Redox Couple in P2-Layered $Na_{0.66}Co_{0.22}Mn_{0.44}Ti_{0.34}O_2$ Cathode for Sodium-Ion Batteries

Qin-Chao Wang, Enyuan Hu, Yang Pan, Na Xiao, Fan Hong, Zheng-Wen Fu, Xiao-Jing Wu, Seong-Min Bak, Xiao-Qing Yang,* and Yong-Ning Zhou**

WILEY-VCH

Copyright WILEY-VCH Verlag GmbH & Co. KGaA, 69469 Weinheim, Germany, 2016.

Supporting Information

Utilizing Co^{2+}/Co^{3+} Redox Couple in P2-layered $Na_{0.66}Co_{0.22}Mn_{0.44}Ti_{0.34}O_2$ Cathode for Sodium-Ion Batteries

Qin-Chao Wang, Enyuan Hu, Yang Pan, Na Xiao, Fan Hong, Zheng-Wen Fu, Xiao-Jing Wu, Seong-Min Bak, Xiao-Qing Yang* and Yong-Ning Zhou**

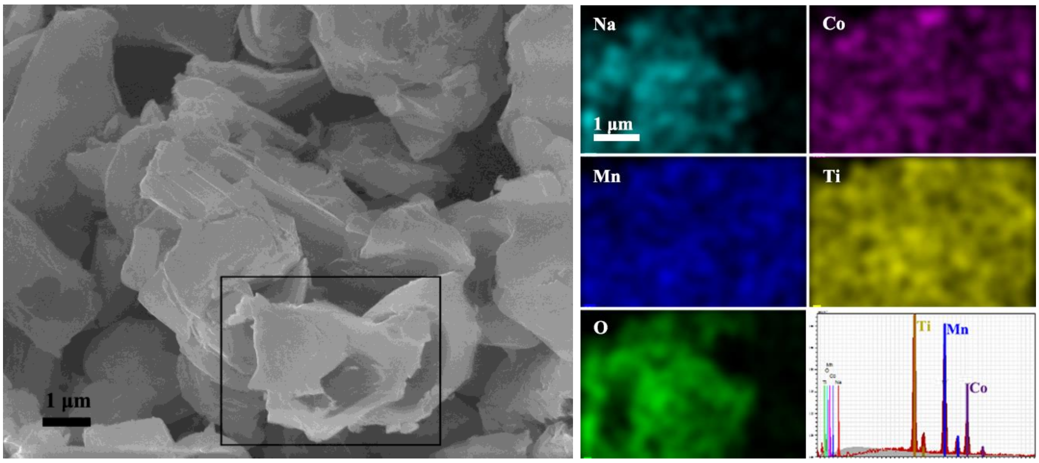

Figure S1. The SEM image of NCMT-2 and corresponding EDX mapping of element Na, Co, Mn, Ti and O.

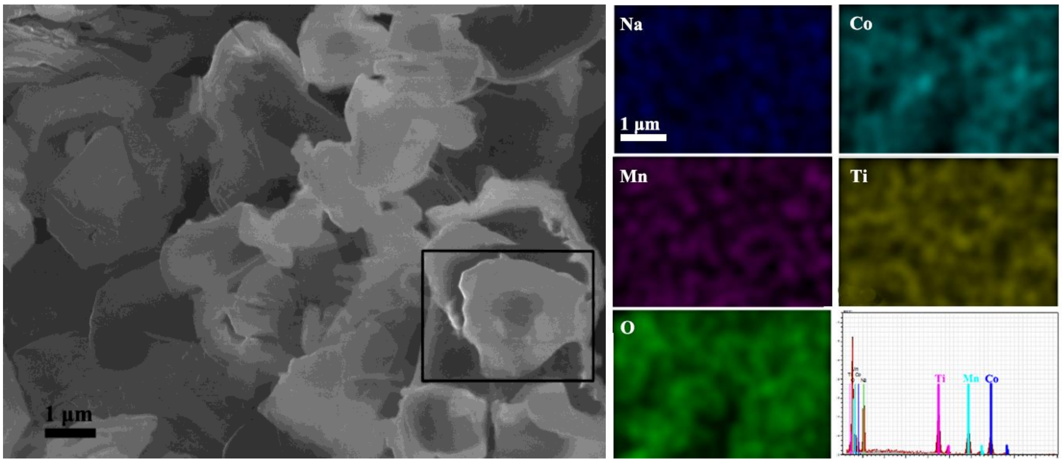

Figure S2. The SEM image of NCMT-3 and corresponding EDX mapping of element Na, Co, Mn, Ti and O.

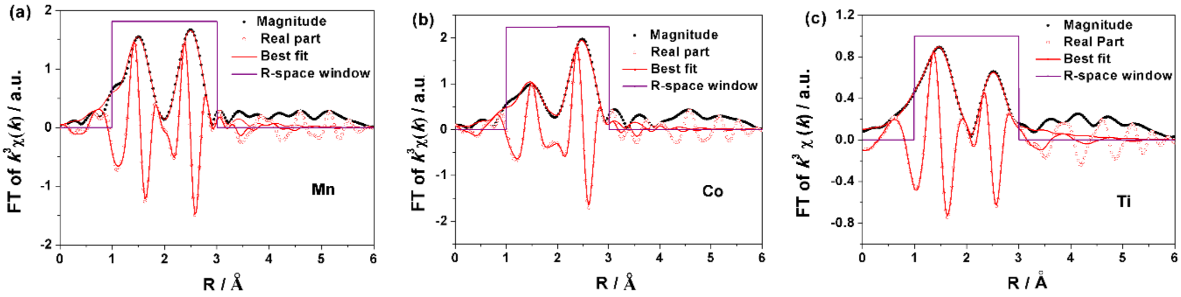

Figure S3. Least-square fits of the calculated FT-EXAFS phase and amplitude functions to the experimental EXAFS spectra for Mn a), Co b) and Ti c) in NCMT-2.

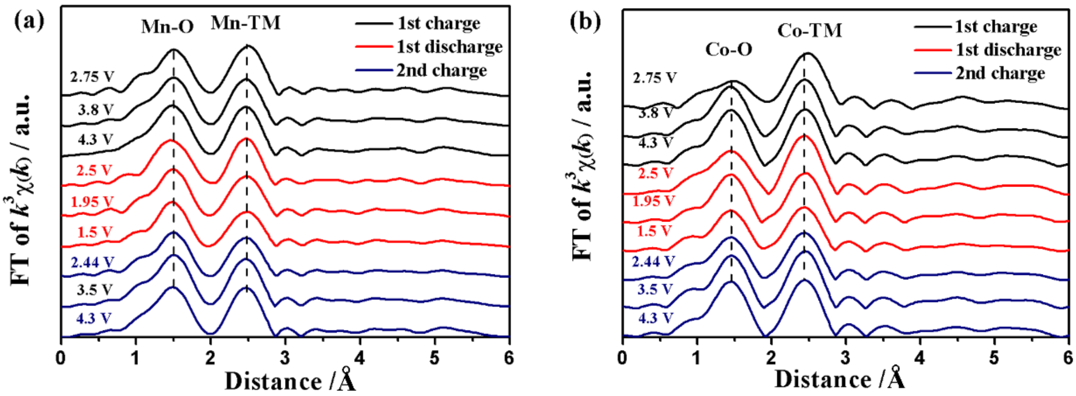

Figure S4. Mn K-edge (a) and Co K-edge (b) Fourier transform magnitudes of k^3 weighted EXAFS spectra of the NCMT-2 at various stages during the first charge (black), first discharge (red) and second charge (navy).

Table S1. The contents of Co, Mn and Ti in NCMT-2 and NCMT-3 evaluated by EDX.

Sample	Element	Series	Atom [at %]	Error [%]
NCMT-2	Co	K-series	24.63	1.2
	Mn	K-series	44.16	1.7
	Ti	K-series	31.21	1.1
NCMT-3	Co	K-series	35.79	1.1
	Mn	K-series	37.23	0.9
	Ti	K-series	35.86	0.7

Table S2. Mn and Co K-edge EXAFS structural parameters of the pristine NCMT-2.

(coordination numbers of Mn-O and Co-O are both 6)

Samples	Path	*r*/Å	σ^2 /10^{-3}Å^2	*ΔE*/eV	*R*
NCMT-2	Mn-O	1.90(5) ± 0.010	2.74 ± 1.49	-4.51 ± 1.51	0.004
	Mn-TM	2.91(0) ± 0.011	3.72 ± 1.25		
	Co-O	2.01(1) ± 0.016	14.83 ± 3.08	-3.47 ± 1.36	0.002
	Co-TM	2.97(4) ± 0.011	6.86 ± 1.48		
	Ti-O	1.89(9) ± 0.006	12.14 ± 1.03	7.48 ± 0.74	0.001
	Ti-TM	2.83(5) ± 0.009	12.99 ± 0.68	-3.27 ± 1.18	

r: bond length; σ^2*:* Debye-Waller factor (disorder); *ΔE:* inner shell potential shift; *R:* R-factor.

Table S3. Mn K-edge EXAFS structural parameters of NCMT-2 at different charge states. (coordination number of Mn-O is 6)

Samples	Path	*r*/Å	σ^2 /10^{-3}Å^2	*ΔE*/eV	*R*
pristine	Mn-O	1.90(5) ± 0.010	2.74 ± 1.49	-4.51 ± 1.51	0.004
	Mn-TM	2.91(0) ± 0.011	3.72 ± 1.25		
charged to 4.3 V	Mn-O	1.89(7) ± 0.009	1.95 ± 1.49	-4.46 ± 1.64	0.004
	Mn-TM	2.88(9) ± 0.011	3.81 ± 1.35		
discharged to 1.5 V	Mn-O	1.91(7) ± 0.007	1.07 ± 1.09	-4.19 ± 1.37	0.004
	Mn-TM	2.91(3) ± 0.010	4.63 ± 1.16		
recharged to 4.3 V	Mn-O	1.89(7) ± 0.010	2.54 ± 1.63	-4.50 ± 1.71	0.005
	Mn-TM	2.88(8) ± 0.012	4.23 ± 1.46		

r: bond length; σ^2: Debye-Waller factor (disorder); *ΔE*: inner shell potential shift; *R*: *R*-factor.

Table S4. Co K-edge EXAFS structure parameters of NCMT-2 at different charge states. (coordination number of Co-O is 6)

Samples	Path	*r*/Å	σ^2 /10^{-3}Å^2	*ΔE*/eV	*R*
pristine	Co-O	2.01(1) ± 0.016	14.83 ± 3.08	-3.47 ± 1.36	0.002
	Co-TM	2.97(4) ± 0.011	6.86 ± 1.48		
charged to 4.3 V	Co-O	1.93(1) ± 0.006	2.70 ± 0.91	-5.29 ± 0.89	0.001
	Co-TM	2.88(5) ± 0.006	4.05 ± 0.75		
discharged to 1.5 V	Co-O	1.98(3) ± 0.008	4.37 ± 1.45	-5.81 ± 1.24	0.003
	Co-TM	2.94(7) ± 0.009	5.12 ± 1.24		
recharged to 4.3 V	Co-O	1.92(9) ± 0.009	2.84 ± 1.41	-5.53 ± 1.24	0.001
	Co-TM	2.88(1) ± 0.010	4.25 ± 1.17		

r: bond length; σ^2: Debye-Waller factor (disorder); *ΔE*: inner shell potential shift; *R*: *R*-factor.
